# Extracellular Vesicles as Therapeutic and Diagnostic Tools for Chronic Liver Diseases

**DOI:** 10.3390/biomedicines11102808

**Published:** 2023-10-17

**Authors:** Aleksandra Leszczynska, Christian Stoess, Hana Sung, Davide Povero, Akiko Eguchi, Ariel Feldstein

**Affiliations:** 1 Department of Pediatrics, University of California, San Diego, CA 92037, USA; aleszczynska@health.ucsd.edu (A.L.);; 2 Department of Surgery, TUM School of Medicine, Technical University of Munich, 81675 Munich, Germany; 3 Department of Biochemistry and Molecular Biology, Mayo Clinic, Rochester, MN 55905, USA; povero.davide@mayo.edu; 4 Biobank Center, Mie University Hospital, Tsu 514-8507, Japan; akieguchi@med.mie-u.ac.jp

**Keywords:** extracellular vesicles, liquid biopsy, biomarkers, therapeutics, liver inflammation, liver fibrosis

## Abstract

Chronic liver diseases can lead to fibrotic changes that may progress to the development of cirrhosis, which poses a significant risk for morbidity and increased mortality rates. Metabolic dysfunction-associated steatotic liver disease (MASLD), alcohol-associated liver disease (ALD), and viral hepatitis are prevalent liver diseases that may lead to cirrhosis. The advanced stages of cirrhosis can be further complicated by cancer development or end-stage liver disease and liver failure. Hence, early detection and diagnosis of liver fibrosis is crucial for preventing the progression to cirrhosis and improving patient outcomes. Traditionally, invasive liver biopsy has been considered the gold standard for diagnosing and staging liver fibrosis. In the last decade, research has focused on non-invasive methods, known as liquid biopsies, which involve the identification of disease-specific biomarkers in human fluids, such as blood. Among these alternative approaches, extracellular vesicles (EVs) have emerged as promising diagnostic and therapeutic tools for various diseases, including chronic liver diseases. EVs are released from stressed or damaged cells and can be isolated and quantified. Moreover, EVs facilitate cell-to-cell communication by transporting various cargo, and they have shown the potential to reduce the expression of profibrogenic markers, making them appealing tools for novel anti-fibrotic treatments. This review focuses on the impact of EVs in chronic liver diseases and exploring their potential applications in innovative therapeutic and diagnostic approaches.

## 1. Introduction

Metabolic dysfunction-associated steatotic liver disease (MASLD) and alcohol-associated liver disease (ALD) are currently two of the most common causes of advanced liver disease worldwide, and their disease spectrum ranges from isolated steatosis to steatohepatitis to cirrhosis. Cirrhosis development is further complicated by an increased risk of hepatocellular cancer (HCC) and chronic liver failure. Unlike steatotic liver disease (SLD), which is defined by liver steatosis, metabolic dysfunction-associated steatohepatitis (MASH) is defined by the presence of both steatosis and lobular inflammation, as well as hepatocyte ballooning degeneration and can be accompanied by fibrosis [1]. The underlying causes and processes for MASLD development and progression are complicated and multifaceted.

MASH is usually asymptomatic until it advances to end-stage liver disease. As a result, early diagnosis of MASLD is crucial to prevent disease progression. The gold standard for diagnosing MASLD is liver biopsy. However, this procedure is invasive and associated with a risk of bleeding and sampling error. Hence, alternative approaches have emerged, avoiding the risk of an invasive procedure. Liquid biopsies, including circulating extracellular vesicles (EVs), can be applied to diagnose diseases based on their molecular signature and properties [2]. Isolation and detection of circulating EVs have been assessed in essentially all body fluids, but plasma and serum are currently the most widely used for discovery and validation of EVs as disease biomarkers.

EVs, which are important mediators of intercellular communication, are necessary for cellular homeostasis and physiological processes, but alterations to these mechanisms may contribute to pathogenic situations. The diagnostic potential of EVs in chronic liver diseases results from their various cargo that changes during disease stages, allowing for the monitoring of disease progression. Additionally, EVs, whether they are unmodified or engineered, have drawn significant attention for their potential as a novel therapeutic modality. EVs are accessible, biocompatible, and resistant to RNases and proteases; therefore, these enzymes have little impact on them. They are suitable delivery systems for drugs, proteins, miRNAs, silencing RNAs, and other small compounds because of their characteristics.

Efforts to shed light on how certain stress signals and molecular responses lead to profibrogenic effects in chronic liver diseases and how these mechanisms can be modulated through targeted therapeutics, such as EVs, are important to improve the prognosis for patients. This review summarizes recent advances in EV research related to chronic liver diseases.

## 2. General Concepts of EVs

Intercellular communication was once considered to be only mediated by direct cell-to-cell contact or the release of soluble substances. It is currently widely accepted that cells may release multiple forms of membrane vesicles in an evolutionarily conserved way as a third sort of cellular interactome.

**Figure 1:**
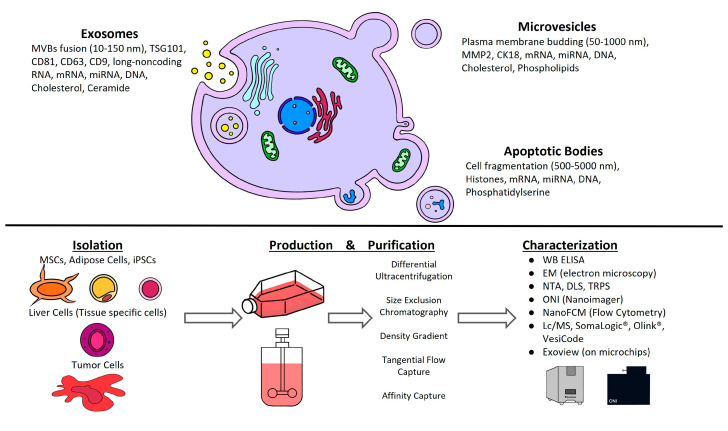
Key steps in EV classification and characterization processes for therapeutic application. Different subtypes of EVs can be classified based on their size and biogenesis as: exosomes, microvesicles, and apoptotic bodies. Isolation, production, purification, and characterization schematic represents the strategic approach to achieve a scalable and optimal workflow for functional EVs.

EVs are a diverse population of cell-released, nanometer-sized vesicles surrounded by a lipid bilayer membrane. Based on their size and cellular biogenesis, EVs are divided into three primary categories: exosomes, microvesicles, and apoptotic bodies. Exosomes (30–150 nm) are produced as intraluminal vesicles within multivesicular bodies (MVBs) and are released after fusing with the plasma membrane. Microvesicles (50–1000 nm) and apoptotic bodies (100–5000 nm) are bigger and produce outward budding and fission with the plasma membrane, or by blebbing during apoptosis, respectively (Figure 1). Biogenesis occurs at different sites of the cell. EVs have similar shapes and sizes and develop using the same intracellular machinery [3]. The cargo sorting process is highly controlled, although the specific processes involved are mostly unknown. As a result, depending on cell physiological or pathological condition, each cell type may regulate EV production quantitatively and qualitatively. Furthermore, if several pathways with distinct activators are implicated, the same cell type may produce diverse populations of EVs.

It is essential to thoroughly characterize EVs. The criteria described in Minimal information for studies of extracellular vesicles 2018 (MISEV2018): a position statement of the International Society for Extracellular Vesicles and update of the MISEV2014 guidelines (MISEV2018) cover the basic categorization for EVs in clinical settings [4]. The characterization methods were carefully evaluated to serve as a guideline. MISEV suggests that isolated EVs should be positive for three transmembrane/lipid-bound proteins (e.g., CD63, CD9, and CD81) and negative for cytosolic protein (e.g., TSG101 and ALIX). The general techniques to characterize EVs are ELISA, Western blot, and imaging techniques, such as Cryo-EM and electron microscopy (EM) [5], and a new method involving the capturing of the EVs on microchips by ExoView (Leprechaun by Unchained Labs) (Figure 1) or super-resolution microscopy ONI Nanoimager. For biophysical analysis NTA (nanoparticle tracking analysis), tunable resistance pulse sensing (TRPS), dynamic light scattering (DLS), NanoFCM Inc, or flow cytometry are used.

## 3. Opportunities in Development of EV Therapeutics in Liver Disease

The vast majority of systemically administered EVs accumulate quickly in the liver, making it the ideal target for EV-based treatments. Notably, the presence of liver damage further enhances this predilection. Considering these properties, EV treatment is particularly promising for liver disease indications.

EVs secreted by mesenchymal stem cells (MSCs) (MSC-EVs) have immunomodulatory properties, making them particularly promising therapeutic avenue for graft-versus-host disease (GVHD). It has been reported that patients who received MSCs-EV treatment had reduced symptoms of GVHD by dampening NK cell response and reducing circulating leukocytes [6]. This suggests that EVs might be used ex vivo for the preconditioning of transplant organs, employing perfusion-based methods in addition to their in vivo use [6]. Furthermore, MSC-EVs have a lower immunogenicity than synthetic EVs and are able to transport their bioactive molecules, including mRNA, miRNAs, immunomodulators, and growth factors, to specific target cells, which makes them the best natural vesicles for precision medicine [7]. The inherent flexibility of EVs to modify their membrane significantly enhances their utility to customize therapies for an individual during treatment [8]. By understanding the functional aspects of EVs, we can gain a better overview of their role in both healthy and diseased states.

**Figure 2:**
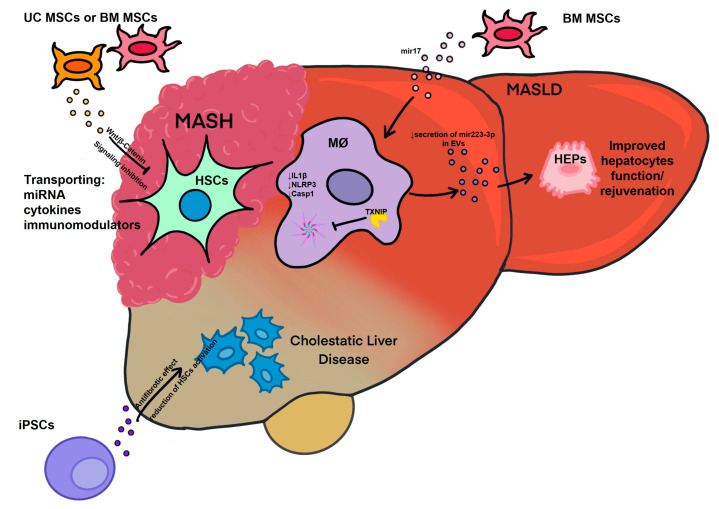
EV function in liver therapeutics. Therapeutic EVs can transport EV cargo to liver cells, alleviating inflammatory or fibrogenic processes and encouraging liver cell repair and rejuvenation, thus contributing to the mitigation of cholestasis, MASLD and their more aggressive forms, MASH, ASH, cirrhosis, or HCC. Abbreviations: MØ—macrophages, HSCs—Hepatic Stellate Cells, Heps—Hepatocytes, UC-/BM MSCs—Umbilical Cord/Bone Marrow Mesenchymal Stem Cells, iPSCs—Induced Pluripotent Stem Cells.

Jiang et al. discovered that exosomes produced from human umbilical-cord-derived MSCs (UC-MSCs) might reduce acute liver damage and fibrosis caused by carbon tetrachloride (CCl4) in mice models by acting as antioxidants [9,10]. Additionally, Rong et al. utilized exosomes derived from human bone marrow MSCs (BM-MSCs) in the therapy of CCl4-induced liver fibrosis via mitigation of HSC activation through the Wnt/β-Catenin pathway [11]. It has been also reported that BM-MSC-derived exosomes may attenuate the hepatic inflammatory response and reduce the release of inflammatory cytokines from macrophages, which may be linked to macrophage expression levels of miR-223-3p and STAT3. In line with this, the treatment with miR-223-3p has shown inhibitory effects on the activation of hepatic stellate cells and improved the development of fibrosis in mice with fibrotic MASH [12]. Furthermore, miR-223-3p has been found to negatively regulate the activation of the NLRP3 inflammasome, a complex involved in inflammatory responses. This regulation occurs through the downregulation of NLRP3 expression, IL-1β production, and the activation of caspase-1. These effects have been observed in both endotoxin acute hepatitis and fibrotic MASH [12]. In a mouse model of hepatic failure induced by the intraperitoneal administration of tumor necrosis factor alpha (TNF-α) and D-galactosamine, the administration of BM-MSC-derived EVs resulted in the infiltration of protective immune cells into the liver [13]. Moreover, Povero et al. highlighted the involvement of EVs derived from induced pluripotent stem cells (iPSCs) in improving liver function. The study demonstrated that iPSC-derived EVs effectively reduced the activation of hepatic stellate cells and liver fibrosis in mouse models of liver fibrosis induced by carbon tetrachloride administration or bile duct ligation [1]. Hou et al. proposed that increased IL-6 potentially works in tandem with fatty acids in MASH to cause myeloid cells to release exosomes. Even though blood IL-6 and miR-223 levels were higher in MASH patients, miR-223 levels in the liver were lower in human MASH patients. Additionally, miR-17, which is prevalent in exosomes, suppresses the activation of the NLRP3 inflammasome by targeting TXNIP (Figure 2), which lowers the levels of ALT and AST in the blood [14].

Limited clinical trials have been conducted on MSC-EV treatments, with most still in progress. The clinical trial NCT01104220 “Role of Immune System in Obesity-related Inflammation and Cardiometabolic Risk” aims to investigate the mechanism underlying fat storage in and around organs, particularly the liver, and how it impacts patients’ health. To gain insights into these processes, the investigators will be examining signaling between the cells and organs via the isolation of the EVs from various body fluids and tissue. The investigators hope to develop improved treatments for conditions such as diabetes and potentially benefit a broader range of patients.

Furthermore, by investigating the rejuvenation and potential reprogramming of adult hepatocytes into progenitor cells, Belmonte and his team have unveiled a promising avenue for further research on EVs [15,16]. Their innovative approach, exemplified by the novel mouse model Hep-4F (4F refers to four Yamanaka factors: Oct-3/4, Sox2, KLF4, and c-Myc), incorporates the (Alb)-Cre transgene, enabling liver-specific gene regulation. They further utilized LoxP-STOP-LoxP-rtTA-IRES-GFP, that which facilitates the controlled administration of doxycycline (Dox), thereby leading to the excision of the Alb cassette and the subsequent expression of rt-TA GFP in hepatocytes, enabling linage tracing. This novel approach for liver rejuvenation allowed Belmonte’s group to achieve the partial reprograming of adult hepatocytes into the progenitor state. Particularly, reprogramming adult cells into iPSC offers great potential for generating patient-specific cells. Moreover, exploring innovative methods in the field of liver rejuvenation, such as utilizing CRISP-CAS9 gene-editing technology, opens exciting possibilities for correcting or even reprograming liver cells to restore their youthful characteristics. It is important to note that this aspect of therapy falls outside the scope of current discussion.

## 4. EVs as Biomarkers of MASLD, ALD, Cirrhosis, and HCC

### 4.1. EVs in MASLD

MASLD is one of the most common forms of chronic liver disease worldwide and encompasses a disease spectrum ranging from simple steatosis to MASH with various degrees of liver fibrosis to cirrhosis. It is estimated that MASLD affects 30% of the population worldwide [17], with obesity and type 2 diabetes mellitus (T2DM) being the most important concomitant conditions [18]. Non-invasive, reliable, and cost-effective diagnostic and prognostic tools for MASLD are currently urgently needed. The gold standard for diagnosing fibrosis is liver biopsy, which is invasive, costly, and may not accurately reflect the complex histopathological onset of MASLD and its progressive form, MASH. Furthermore, imaging or liver biopsy only offer a partial delineation of the disease progression or stage. Circulating EVs may fill this gap by providing a real-time estimation of disease stage and progression based on EV number, molecular composition (proteins, miRNA, lipids, and mRNA), size, and specific markers of the parental cells (Figure 2). For instance, greater levels of circulating EVs have been extensively described in plasma samples collected from rodents and human subjects with chronic liver diseases compared to healthy controls [19,20,21,22]. One of the first reports by Kornek et al., describing the role of circulating EVs in patients with two different chronic liver diseases, chronic viral hepatitis C (CHC) and MASLD/MASH, identified CD4+ and CD8+ T-cell-derived microparticles (MPs) in the plasma of patients with active CHC and MASLD/MASH [23]. In the same study, the level of activated T-cell-derived circulating MPs correlated with disease severity as determined by alanine aminotransferase (ALT) values and exhibited fibrolytic properties. Notably, MPs released from activated or apoptotic human T cells in vitro were capable of fusing with hepatic stellate cells, where they unloaded a variety of membrane molecules, such as CD147, which triggered fibrolytic responses. The ability to communicate with the target cells is distinctive of EVs and can occur at the cell surface level, either in a membrane-receptor-dependent or -independent manner, and it is followed by internalization or membrane fusion [24]. The transfer of EV cargo has been reported to occur via clathrin-mediated endocytosis, caveolin-dependent mechanisms, macropinocytosis, lipid rafts, or phagocytosis, as extensively described elsewhere [24,25]. However, information on biologically circulating MP-triggered HSC pro-fibrogenic activation remains limited. More recent reports have explored this aspect of EVs by providing further insights on the specific bioactive molecules involved in EV-mediated HSC activation and fibrosis and by uncovering some of the pathways activated by EVs upon uptake by target cells, as elegantly described elsewhere [26,27,28,29]. The correlation between circulating EV levels and disease severity was confirmed in independent murine experimental models of MASLD and MASH [30]. In an earlier study, it was reported that circulating EV count increased with MASLD progression and correlated with liver fibrosis, injury, and pathological angiogenesis in diet-induced murine MASLD [30]. In addition, it was reported that circulating EVs identified in advanced experimental murine MASH were enriched in miR-122 and miR-192, two miRNAs abundantly expressed in hepatocytes. This study also identified the proteomic profile of circulating EVs released in advanced MASH mice compared to those released by the control animals. Notably, a significant number of differentially expressed proteins were identified between the two experimental groups and included proteins involved in cell death, angiogenesis, redox homeostasis, and inflammation [30]. In a subsequent study, circulating EVs were further characterized over time in a different diet-induced experimental model of MASH, both in female and male mice, with the goal of comparing laboratory-based findings with the established biomarkers of histology [31]. This study further corroborated the evidence that EV origin could be determined among total circulating EVs in plasma samples. Indeed, both hepatocyte- and non-parenchymal-cell-derived circulating EVs were increased in MASH male and female mice in a time-dependent manner. Specifically, levels of hepatocyte-derived circulating EVs, identified among total circulating EVs as those expressing either asialoglycoprotein receptor 1 (ASGR1) or Cytochrome (CYP)2E1, correlated with hepatic ballooning, inflammation, and fibrosis. Overall, this report demonstrated that hepatocyte- and immune-cell-derived circulating EVs correlate with histological assessment of MASH and non-invasive magnetic resonance-based biomarkers of MASH [31]. These studies have also shed light on the detection of hepatocyte-specific circulating EVs, which account for about 20% of the total EVs and whose isolation is frequently challenging. To overcome this challenge, a recent report evaluated a novel tool for the detection of hepatocyte-specific circulating EVs. Plasma EVs isolated from patients with MASH pre- and post-weight loss were used for the far-field nano-plasmonic enhanced scattering (nPES) assay, an antibody-based system designed to capture hepatocyte-specific circulating EVs based on specificity for hepatocyte transmembrane proteins ASGR2 and CYP2E1. Strikingly, the nPES assay accurately identified ASGR2+ and CYP2E1+ circulating EVs, which were also positive for the EV marker CD63. Strikingly, the data of this report showed that circulating hepatocyte-derived EVs are elevated in MASLD patients and significantly decrease upon weight loss surgery and provide support for potential use of nPES as a point-of-care biomarker for prognostic and diagnostic purposes [22]. The circulating EV content has been further explored over the years in human MASLD, with the goal of validating some of the earlier murine experimental studies. Various studies have described the proteomic, miRNA and lipidomics profiles of circulating EVs in patients with MASLD. In the paper by Nakao et al. [22], small circulating EVs isolated from MASLD patients pre- and post-weight loss surgery were significantly depleted in diacylglycerol, triacylglycerol, and cholesterol esters but were enriched in sphingolipids, particularly sphingolipid-1-phosphate, long-chain ceramides, and dihydroceramides, compared to large EVs. In a different independent report, circulating EVs were also reported to increase in advanced MASH patients compared to the healthy controls and increased even further in patients with MASH cirrhosis compared to pre-cirrhotic MASH patients. As shown in previous reports, this study also confirmed the strong association between the levels of hepatocyte-derived circulating EVs and clinical characteristics of MASH, including fibrosis stage. According to the findings of this report, proteomics analysis of circulating EV cargo identified unique protein signatures in each of the three study cohorts, which showed strong prognostic power [20]. However, the currently available studies would need to be further validated in larger study populations and include EVs isolated from disease control groups.

### 4.2. EVs in ALD

ALD is one of the most prevalent subtypes of SLD and its pathological progression ranges from simple steatosis to steatohepatitis to cirrhosis. The most common form of ALD is alcohol-associated steatohepatitis (ASH), which is associated with severe inflammation [32,33]. Similarly to MASLD, diagnosis of ALD still relies significantly on the clinicopathological assessment of liver biopsies or imaging findings. In addition, non-invasive biological serum tests are available, namely, non-patented methods, such as APRI (AST to platelet ratio index) and fibrosis-4 (FIB-4), or patented methods, such as FibroTest and the enhanced liver fibrosis (ELF) test. Further development of inexpensive yet robust and reliable biomarkers for ALD is urgently needed. In recent years, various reports have explored the hypothesis that circulating EVs may add to the existing repertoire of ALD biomarkers. The first evidence of the potential application of circulating EVs as ALD biomarkers originated from a report by Momen-Heravi et al., which showed a strong correlation between exosomes and ALT levels, thus demonstrating how EVs can mirror ethanol-induced hepatocellular damage [34]. The analysis of the genetic content identified the abundant presence of total RNA in EVs compared to EV-free compartments and the enrichment of liver-specific miR-122, miR-30a, and miR-192 in the EVs of alcohol-fed mice. These findings were corroborated in circulating EVs isolated from patients with ASH, which were also enriched in miR-30a and miR-192 but not miR-122, which may suggest physiopathological differences between the alcohol animal model and patients with ASH [34]. A study from our group, confirmed the significant presence of circulating EVs in an experimental murine model of ASH [35,36,37]. The isolated EVs expressed miRNA signatures that may provide disease-specific “barcodes” for the non-invasive diagnosis of ASH. Overall, the findings strongly point to the significant clinical relevance of total circulating EVs and distinct miRNA “barcode” for the diagnosis of ALD and for the distinction of ASH from SLD. The findings were validated in a small study population of ALD patients and controls, where not only EV levels correlated with disease severity but also contained the miRNA barcode previously identified in a murine model of ALD [21]. A larger human study conducted by Sehrawat and colleagues in 36 patients with heavy alcohol consumption and 36 patients with confirmed ASH identified an EV number cutoff for the accurate diagnosis of ASH compared to patients with heavy alcohol consumption, healthy controls, and MASH. Notably, ASH EVs were enriched in sphingolipids, which did not predict ASH severity and mortality, but the combination of EV counts and sphingolipid species increased the performance of the MELD score in predicting 90-day mortality [21]. Despite the significant progress of the EV characterization and validation as the biomarkers of liver diseases, such as ALD, further studies using larger sample sizes and multi-omics strategies will uncover novel aspects of circulating EVs and will enhance current circulating EV detection methods [38].

### 4.3. EVs in Cirrhosis

Liver cirrhosis is the final stage of hepatic fibrogenesis. It affects 1–2% of the world’s population and leads to more than one million disease-related deaths annually worldwide [39,40]. The rather multi-dynamic process of fibrotic remodeling is influenced by many different key factors of intercellular communication. In this respect, EVs are released from different cell types, which determines these vesicles to serve as an attractive source of tissue-specific biomarkers that would help to monitor the disease and thereby exclude the risks of an invasive liver biopsy. Furthermore, the measurement of both the quality and quantity of EVs enables the delineation of distinct disease stages, thereby presenting an additional avenue for prognostic evaluation. Audrey Payance et al. showed in their large prospective study with 139 patients (advanced fibrosis *n* = 10 and cirrhosis *n* = 129) that circulating hepatocyte MP values can predict the 6-month mortality rate in patients with cirrhosis, independently of established scores, such as the Child–Pugh score or MELD. Patients with hepatocyte MP values >65 U/L and MELD >15 had a significantly higher 6-month mortality than other patients (23% versus 3%). These results were confirmed in a validation cohort of 103 patients [41]. In another study by Engelmann et al., ascites- and blood-derived MPs also appeared to play a role in the setting of end-stage liver disease and decompensated cirrhosis [42]. Ascites and blood samples of 163 patients with cirrhosis (ascites *n* = 163 and blood *n* = 31) were collected. EVs were detected in all samples. Interestingly, an inverse correlation of EV levels in ascites were seen as the levels decreased in patients who did not survive, with increased levels of a CD66b–CD3-positive subset of EVs. In patients who died or were transplanted within 30 days after paracentesis, ascites MP levels were significantly lower (median of 180.5 (32.5–2851.6) MP/μL) as compared to patients that survived this period (325.1 (17.5–32,575.1) MP/μL). The decreased MPs in ascites showed no correlation with other clinical prognostic factors (such as the Child–Pugh score). In line with these findings, Sanchez-Rodriguez et al. observed decreased levels of EVs in patients with compensated cirrhosis (*n* = 6) as well as in acute decompensation (*n* = 11) and acute-on-chronic liver failure (ACLF, *n* = 11). However, EVs were not characterized for hepatocyte-specific markers. Nevertheless, the results of this study added knowledge to the involvement of EVs in the dynamic process of the inflammatory state of the liver during the progression of cirrhosis and deterioration of liver function [43]. With respect to utilizing EVs as biomarkers in cirrhosis, there are contradictory findings regarding their absolute levels. While most studies indicate an elevation in EVs derived from hepatocytes during the advanced stages of chronic liver disease, the studies above indicate more complex and intricate dynamics [19,21,41,44]. Not only are the absolute levels of EVs in patients of high interest, but also identifying subsets of MPs in cirrhotic patients is a central issue and only scarce data are available. To address this issue, Rautou and colleagues performed a study where MPs from the blood samples of 91 patients with cirrhosis and 30 healthy individuals as the controls were analyzed by flow cytometry. The circulating levels of leuko-endothelial (CD31+/41-), pan-leukocyte (CD11a+), lymphocyte (CD4+), and erythrocyte (CD235a+) MPs were higher in patients with cirrhosis than those in the controls. MP levels were overall significantly associated with survival, independent of the Child–Pugh score but not of the MELD score [45]. From a clinical perspective, it must be noted that it is difficult to obtain samples of sufficient quality and quantity from most critically ill patients with end-stage liver disease and to monitor them over a specific period. In conclusion, EVs show a potential utility as biomarkers in severe disease in cirrhotic patients, but further studies are needed to validate the findings and to better understand conflicting results.

### 4.4. EVs in Hepatocellular Carcinoma

Liver cancer is a primary cause of mortality globally. The number of new cases and fatalities from liver cancer is anticipated to increase by more than 55% by 2040 [46], owing to lifestyle changes and the impact of MASLD. HCC is the most common primary liver cancer accounting for approximately 75% of the total. It is desirable to develop specific diagnostic markers to improve diagnostics and the early detection of tumor recurrence. At present, single tumor markers, such as AFP (for HCC) and CA19-9 (CCA), are very standardized but rather unspecific [47]. Recent developments, however still in an experimental setting, have led to the discovery of various potential new biomarkers that could detect liver tumors, such as circulating tumor cells, cell-free DNA/RNA, miRNA, and EVs. By avoiding the risks of a liver biopsy, the concept of liquid biopsy becomes increasingly important due to its high safety [48,49,50]. In the context of this concept, EVs are of growing interest. Not only are they released by tumor cells and can promote cancer stem cell formation and tumorigenesis, but it is also anticipated that a unique EVs barcode can lead to the accurate detection of early tumor development and even better differentiation between different tumor entities, such as HCC, CCA, and liver metastases [51]. Abbate et al. conducted a pilot study with 15 HCC patients (controls with cirrhosis *n* = 5 and healthy controls *n* = 5), which showed that the blood levels of HepPar1+ MPs in patients undergoing surgical resection for HCC had a prognostic impact. The levels of HepPar1+ MPs were higher before surgery and increased at the time of tumor recurrence. Interestingly, HepPar1+ MPs were virtually absent in the blood circulation of cirrhotic patients without HCC and the healthy controls. These data are important, since they indicate that circulating HepPar1+ MPs are tumor-specific and may have the potential to serve as specific biomarkers for the diagnosis and recurrence of HCC [52]. Not only are the total levels of EVs and their respective cell-specific markers of particular interest, but also the exosomal cargo itself. Johann von Felden and colleagues isolated EVs from 375 patients with either HCC or prostate cancer. Briefly, they characterized a 3-smRC signature (small RNA clusters) based on extracellular unannotated RNAs, which was significantly overexpressed in the plasma of patients with HCC. An independent validation in a phase 2 biomarker case–control study revealed 86% sensitivity and 91% specificity for the detection of early HCC from the controls at risk (AUC: 0.87) [53]. Another cargo of interest are tRNA-derived small RNAs (tsRNAs), which are usually 18–40 nucleotides in length and generated from precursor or mature tRNAs. The results of a small study with liver cancer patients showed a significantly higher abundance of a distinct set of different tsRNAs [54]. The combination of exosomal miR-10b-5p + miR-221-3p + miR-223-3p + miR-21-5p in liver-specific exosomes was shown to perform well in distinguishing HCC from patients with chronic viral hepatitis (AUC: 0.86) with an even higher sensitivity in a combination of miR-10b-5p + miR-221-3p + miR-223-3p (AUC: 0.84) for low AFP-HCC vs. chronic hepatitis/non-HCC patients [55]. The development of HCC is linked to compromised autophagy, resulting in the increased production of extracellular vesicles, such as exosomes and microvesicles [56,57]. Recent studies revealed complex associations between exosome formation and autophagy, where autophagy machinery can either boost or hinder EV secretion [58]. The modulation of autophagy can significantly influence both the quantity and content of EVs, consequently playing a pivotal role in determining whether autophagy modulators have pro-tumorigenic or anticancer effects. Recent research has implicated autophagy regulators in the unconventional secretion of proteins via exosomes [59]. The ATG8 conjugation machinery, for instance, mediates the cargo loading of multiple RNA-binding proteins into EVs through a process referred to as microtubule-associated protein 1A/1B-light chain 3 (LC3) LC3-dependent EV loading and secretion (LDELS) [56]. This mechanism is dependent on LC3 and requires the activation of neutral sphingomyelinase (nSMase-2 or SMPD3), which plays a role in intraluminal budding during EV biogenesis [60]. Furthermore, studies have shown that the pharmacological inhibition of lysosomes can lead to the release of LC3-II and autophagic cargo through EVs and EV-associated secretory intermediates. Lysosomal blockade promotes the extracellular secretion of autophagic cargo receptors (ACRs), including p62, which are released as EV-associated nanoparticles within a subset of extracellular vesicles called extracellular vesicles and particles (EVPs) [59]. This phenomenon is referred to as secretory autophagy during lysosome inhibition (SALI) and requires multiple ATG proteins for autophagosome formation, as well as RAB27A, which plays a key role in the release of vesicles from cells [61]. Importantly, ACRs secreted via SALI have been detected in EVPs isolated from blood plasma following treatment with drugs such as hydroxychloroquine [62]. As a result, measuring the autophagy-dependent EVP secretome in human plasma may serve as a powerful biomarker for non-invasively monitoring the effectiveness of next-generation lysosomal inhibitors in cancer treatment.

## 5. Concluding Remarks and Future Directions

Recent research has shown that EVs can serve as novel biomarkers in various liver diseases. Furthermore, their ability to safely transport cargo between cells may enhance the cell-specific delivery of various synthetic and biological compounds for the treatment of liver disease. Before controlled clinical trials can be conducted, many significant difficulties, such as the selection of the specific EV type for the administration and standardization of techniques, must be overcome. However, due to the complexities of these nano-vesicles, the majority of potential diagnostic and therapeutic methods are still in the preclinical or early clinical stages. In addition, comprehensive examinations of cell/tissue-specific molecular patterns that are conducted by EVs are needed. The identification of distinct molecular signatures of released EVs is compelling, but more research is needed to rigorously test these markers in larger patient cohorts. Once this is accomplished, useful and prospective diagnostic and therapeutic tools can be developed.
